# Nuclear Legumain Activity in Colorectal Cancer

**DOI:** 10.1371/journal.pone.0052980

**Published:** 2013-01-10

**Authors:** Mads H. Haugen, Harald T. Johansen, Solveig J. Pettersen, Rigmor Solberg, Klaudia Brix, Kjersti Flatmark, Gunhild M. Maelandsmo

**Affiliations:** 1 Department of Tumor Biology, Institute for Cancer Research, Oslo University Hospital – The Norwegian Radium Hospital, Oslo, Norway; 2 School of Engineering and Science, Research Center MOLIFE – Molecular Life Science, Jacobs University Bremen, Bremen, Germany; 3 Department of Pharmaceutical Biosciences, School of Pharmacy, University of Oslo, Oslo, Norway; 4 Department of Gastrointestinal Surgery, Oslo University Hospital – The Norwegian Radium Hospital, Oslo, Norway; 5 Department of Pharmacy, Faculty of Health Sciences, University of Tromsø, Tromsø, Norway; Stanford University, United States of America

## Abstract

The cysteine protease legumain is involved in several biological and pathological processes, and the protease has been found over-expressed and associated with an invasive and metastatic phenotype in a number of solid tumors. Consequently, legumain has been proposed as a prognostic marker for certain cancers, and a potential therapeutic target. Nevertheless, details on how legumain advances malignant progression along with regulation of its proteolytic activity are unclear. In the present work, legumain expression was examined in colorectal cancer cell lines. Substantial differences in amounts of pro- and active legumain forms, along with distinct intracellular distribution patterns, were observed in HCT116 and SW620 cells and corresponding subcutaneous xenografts. Legumain is thought to be located and processed towards its active form primarily in the endo-lysosomes; however, the subcellular distribution remains largely unexplored. By analyzing subcellular fractions, a proteolytically active form of legumain was found in the nucleus of both cell lines, in addition to the canonical endo-lysosomal residency. *In situ* analyses of legumain expression and activity confirmed the endo-lysosomal and nuclear localizations in cultured cells and, importantly, also in sections from xenografts and biopsies from colorectal cancer patients. In the HCT116 and SW620 cell lines nuclear legumain was found to make up approximately 13% and 17% of the total legumain, respectively. In similarity with previous studies on nuclear variants of related cysteine proteases, legumain was shown to process histone H3.1. The discovery of nuclear localized legumain launches an entirely novel arena of legumain biology and functions in cancer.

## Introduction

Legumain, or AEP (asparaginyl endopeptidase), belongs to the cysteine protease family C13 in the clan CD according to the MEROPS Peptidase Database [1]. It was first discovered in beans [2] and blood fluke (*Schistosoma mansoni*) [3] before Chen and co-workers described the mammalian version in 1997 [4]. The mammalian protease is clearly homologous with legumain from non-mammalian species and the conservation along the evolutionary lineage presumably indicates functional importance. The human pro-enzyme of 433 amino acids undergoes several successive cleavages both N- and C-terminally, of which some require acidic pH, before reaching the mature active enzyme form. The maturation process is partially autocatalytic, but depends also on other proteolytic enzymes which, along with the complete understanding of the activation process, have not been fully characterized [5]–[7]. The active protease shows highly specific preference for substrate hydrolysis C-terminally to asparagine and to some extent after aspartic acid under more acidic conditions. The most potent endogenous inhibitors of legumain are cystatin E/M and cystatin C [8], [9], whereas the classic chemical inhibitor of cysteine proteases, the compound E64, does not affect legumain activity [4].

There are several reports of legumain being over-expressed in a number of solid tumors (e.g. colorectal and breast cancers), and this has also been correlated to a more invasive and metastatic phenotype [10]–[12]. Recently, we screened a panel of melanoma cell lines and found that legumain was expressed and active in most of the cell lines investigated [13]. In normal tissues, legumain is most prominently expressed in the placenta, kidney and spleen [10]. Legumain knock-out mice are born healthy and fertile, but display reduced body weight, aberrant endo-lysosomes with development of kidney failure and extramedullary hematopoiesis in the spleen [14]–[16].

Recently it has been shown that legumain may be involved in cell proliferation independent of the endopeptidase activity [17]. Furthermore, legumain has been demonstrated to activate proMMP-2, which may partially explain the observed association between legumain expression and metastatic potential [18]. The strict substrate specificity combined with over-expression in various tumor types has motivated exploitation of legumain as a pro-drug activator in cancer treatment, for instance by adding a cleavable peptide chain to doxorubicin or auristatin [10], [19] and targeting of drug compounds using a legumain enzyme inhibitor [20]. Other known biological functions of legumain include autophagic-lysosomal processing of hepatocellular proteins [21], processing of antigens for MHC class II presentation [22], and maturation in Toll-like receptor signaling [23].

In this study, legumain expression and proteolytic activity were examined in two colorectal carcinoma (CRC) cell lines, HCT116 and SW620. Remarkable differences in activity were initially identified and we further used these cell lines to examine legumain distribution and activity at subcellular levels. Of great interest and rather unanticipated, nuclear proteolytic active legumain was revealed in both cell lines in addition to the expected endo-lysosomal localization. These results were further acknowledged by immunofluorescence, immunohistochemistry and *in situ* activity measurements in cultivated cells and xenografts, and also documented in human CRC tumor tissue. Finally, legumain was shown to proteolytically cleave histone H3.1 *in vitro* unveiling a potential functional implication of nuclear localized legumain activity.

## Materials and Methods

### Cell lines, xenografts and CRC biopsies

RKO, CO205, SW48, Colo320DM, HT29, SW620 and HCT116 were bought from American Type Culture Collection (ATCC). KM20L2 and HCC2998 (DCTD Tumor/Cell Line Repository) were kindly provided by Dr. Michael R. Boyd (National Cancer Institute, Frederick, MD, USA), as well as LS174T [24] and TC7 [25] cell lines from Dr. Richard Hamelin (INSERM, Paris, France). Cell line identity was validated by short tandem repeat analysis for the HCT116 and SW620 cell lines. Cells were cultivated in RPMI 1640 (BioWhittaker) containing 10% fetal bovine serum (Hyclone), 20 mM Hepes (BioWittaker) and 2 mM Glutamax (Invitrogen). All cell lines were routinely tested negative for *Mycoplasma*. Subcutaneous xenografts from HCT116 and SW620 were established by injection of 1*10^6^ cells in both flanks of locally bred female BALB/c nude (nu/nu) mice [26]. Housing and all procedures involving animals were performed according to protocols approved by the Oslo University Hospital Animal Care and Use Committee, in compliance with the Norwegian Animal Research Authority guidelines on animal welfare. Human biopsies were obtained from patients during primary surgery of assumed or verified CRC. The study was approved by the Regional Ethics Committee of Southern Norway (#S-98080) and written informed consent was obtained from the patients.

### Cell lysates, conditioned media harvesting and subcellular enrichment

To obtain cell lysates for immunoblotting, sub-confluent cultures were detached using EDTA (BioWittaker) and washed 3 times in ice cold PBS (BioWittaker) before cold lysis buffer (150 mM NaCl, 50 mM Tris-HCl pH 7.5, 0.1% NP-40) with the protease inhibitor mixture CompleteMini (Roche Diagnostics) was added to dry cell pellets and left on ice for 15 min. Finally, the samples were sonicated and centrifuged at 15000×g for 15 min to remove cell debris. In samples for activity measurements a lysis buffer (100 mM sodium citrate, 1 mM disodium EDTA, 1% n-octyl-beta-D-glucopyranoside, pH 5.8) without protease inhibitors was used. Protein concentrations were determined using the BCA (bicinchoninic acid) protein assay kit (Pierce) or Bradford assay [27]. All samples were stored at −80°C. Conditioned cell medium was acquired by seeding 7.5*10^5^ cells in 6-well plates and grown overnight in medium containing serum, then washed and grown for another 24 hours in 1 ml serum-free medium. The serum-free conditioned medium was centrifuged at 15000×g for 5 min and the supernatant collected. Proteins from the conditioned medium were concentrated by addition of 4 volumes of ice cold acetone, leaving the samples on ice for 15 min and centrifugation at 4°C and 12000×g for 10 min. Liquid was removed and the precipitate air dried at room temperature before re-dissolving in buffers for immunoblotting or activity measurements, according to subsequent protocols. Subcellular enrichment of lysosomes and nuclei was performed by density gradient centrifugation according to Brix *et al.* [28] with separation of the fractions repeated twice to ensure high purity. Lysis buffers were adjusted to pH 5.0 and 7.4 for lysosomal and nuclear fractions, respectively. All other subcellular enrichment was performed in triplicates using the Subcellular Protein Fractionation Kit for Cultured Cells (Thermo Scientific) on 5*10^6^ HCT116 cells and 10*10^6^ SW620 cells to obtain equal volumes of cell pellets as starting material according to the manufacturers' protocol, and with the addition of washing the pellet between all fractions to ensure high purity.

### Immunoblotting and ELISA

Samples were run on NuPAGE gels 4–12% (Invitrogen) at 150 V and room temperature using the supplied MES-buffer (containing SDS) according to the manufacturers' protocol, and then blotted onto 0.45 µm polyvinylidene fluoride (PVDF) membranes (Millipore Corp.) in the X-cell Sure lock (Invitrogen) at 4°C for one hour in 20% methanol containing Tris-Glycine buffer. Quality of protein transfer was verified using Amidoblack for 1D gels. The membranes were subsequently blocked for one hour at room temperature in 5% dry milk TBST-buffer (Tris-Buffered Saline with Tween 20) and probed with primary antibody in 5% dry milk TBST-buffer for one hour at room temperature at the following concentrations: legumain goat polyclonal antibody (pAb) (1∶1000; R&D Systems; AF2199), cathepsin L goat pAb (1∶500; R&D Systems; AF952), cathepsin B rabbit pAb (1∶10000; Calbiochem; 219408), cystatin E/M goat pAb (1∶500; R&D Systems; AF1286), α-tubulin mouse monoclonal antibody (mAb) (1∶5000; Calbiochem; CP06), arylsulfatase B (ARSB) mouse mAb (1∶500, R&D Systems; MAB4415), lysosomal-associated membrane protein (lamp-2) mouse mAb (1∶250, Santa Cruz, sc-18822), specificity protein 1 (SP1) rabbit pAb (1∶10000, Millipore, 07-645), and histone H3 rabbit mAb (1∶10000, Millipore, 05-928). Then, the membranes were washed 3 times in buffer without dry milk, probed with HRP-secondary (horseradish peroxidase) antibody (1∶5000; DakoCytomatation) specific against corresponding species for one hour at room temperature, and subsequently washed 3 times. Development was performed using SuperSignal West Dura Extended Duration Substrate (Pierce) according to the manufacturers' instructions, visualized on medical X-ray films (Kodak or Thermo Scientific) and converted to TIFF using a flatbed film scanner (Canon). For densitometry analyses the film was scanned in a calibrated densitometer GS-800 (Bio-Rad) and quantified by QuantityOne v.4.6.5 (Bio-Rad). All measured quantities were normalized using the corresponding loading control (α-tubulin). ELISA measurements of human total legumain (R&D Systems, DY4769) from three separate protein isolations were performed in duplicate according to the manufacturers' recommendations.

### Mutation analyses

DNA from the cell lines HCT116 and SW620 was isolated using the QIAamp DNA Blood Mini Kit (Qiagen). *LGMN* (ENSG00000100600) exon 12 (ENSE00000808693) was subsequently generated by PCR using specific forward (5′-agaggctggacttggggtat-3′) and reverse (5′-gcttccgttacatggaggac-3′) primers. Sequencing reactions were performed using the same primers and the Dyenamic ET Dye Terminator Cycle Sequencing Kit (Amersham) as described by the supplier. The samples were finally subjected to post clean up, separated by capillary electrophoresis and analyzed using a MegaBACE1000 sequencing instrument (Amersham).

### Legumain activity, immunofluorescence and immunohistochemistry

Legumain activity in cell lysates and subcellular fractions was measured in triplicate by cleavage of the substrate Z-Ala-Ala-Asn-NHMec (Department of Biochemistry, University of Cambridge, UK) as previously described [4], [29]. In brief, cell lysate (20 µl) was added to black 96-well microplates (No. 3915; Costar, Corning). After the addition of 100 µl buffer and 50 µl substrate solution (final concentration 10 µM) at either pH 5.8 or 7.4, a kinetic measurement based on increase in fluorescence over 10 min was performed at 30°C in a plate reader (Wallac Victor 3, PerkinElmer) and presented as enzyme units where one unit of activity was defined as the amount of enzyme releasing 1.0 µmol of product/min under the standard conditions described. Immunofluorescence was performed on cells grown on sterilized glass slides in 6-well plates subsequently fixed in 4% PFA and permeabilized with 0.2% Triton-X100 before staining with legumain primary antibody (1∶100) and a secondary antibody conjugated with Alexa488 (Invitrogen, 1∶200, A-11034) in buffer containing 0.1% BSA. Control slides were prepared without addition of primary antibody. Nuclei were stained with DRAQ5™ (Biostatus) and coverslips mounted in Mowiol in 200 mM Tris-HCl, pH 8.5 (Hoechst) before observation on laser-scanning confocal imaging system LSM510 or LSM710 (Carl Zeiss). Image arithmetics were performed according to Jedeszko *et al.* [30] using Image J [31]. The analysis of nuclear localized legumain was performed on 5 z-stacks each composed of 25–35 sections and each containing 30–40 cells captured without saturated pixels.

*In situ* activity of legumain in cells and tissue sections from xenografts was measured by cleavage of the substrate Suc-Ala-Ala-Asn-NHNapOME (Department of Biochemistry, University of Cambridge, UK) as previously described and verified on tissue from legumain knock-out mice [32], [33] using final concentrations of 1 mM 5-nitro-salicylaldehyde, 0.5 mM substrate and supplied with DAPI (Invitrogen) to visualize nuclei. Cells mounted in OCT Compound (Tissue-Tek) and xenografts were cut into cryostat sections (6 µm) and incubated with 50 µl assay solution for 10–15 min at 37°C before observation by means of laser-scanning confocal imaging system LSM710 or LSM510, respectively, and using the co-localization module of the Zen 2009 software for pseudo-coloring (white). Control slides were prepared using buffer without substrate, the epoxy inhibitor E64 (Sigma) at a final concentration of 1 µM or human recombinant cystatin E/M (R&D Systems, 1286-PI) at a final concentration of 0.1 µM. Immunohistochemical staining was performed on formalin-fixed, paraffin-embedded tissue sections from subcutaneously grown xenografts and human CRC biopsies, using the legumain antibody at 1∶300 dilution with the biotin-streptavidin-peroxidase method as described previously [34]. Goat-IgG isotype control stainings were performed at similar concentration on xenograft and CRC tumor tissue sections.

### Proteolytic cleavage of histone H3.1

Human recombinant legumain (R&D systems, 2199-CY) was auto-activated at 37°C for 2 h in acidic buffer (50 mM NaOAc, 100 mM NaCl, pH 4.0) at concentration 0.1 µg/µl. Bovine legumain was isolated from kidney as described by Yamane *et al.* [35]. Human recombinant histone H3.1 (New England BioLabs, M2503S) was added to 50 µl assay buffer (50 mM MES, 250 mM NaCl, pH 5.0 or pH 7.0) with or without cystatin E/M and with final addition of either active human or bovine legumain. Each mixture was incubated at 37°C for 2 h with shaking.

## Results

### Legumain and cathepsin L are heterogeneously expressed in CRC cell lines

**Figure 1:**
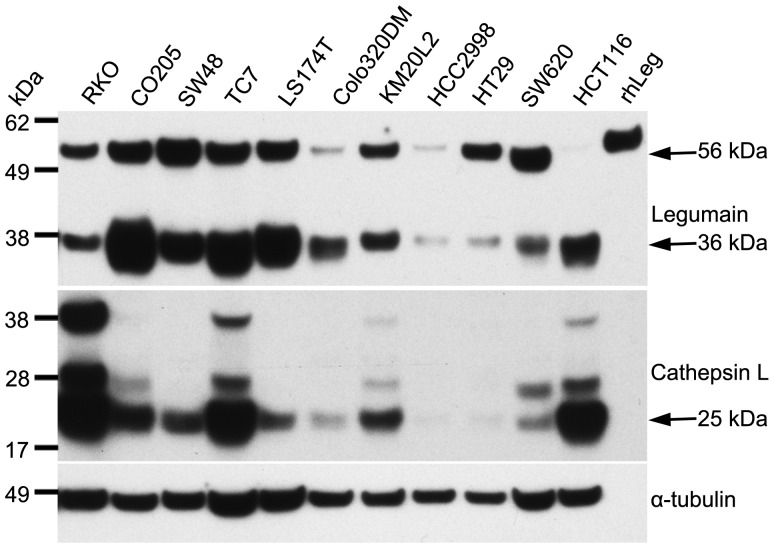
Expression of legumain and cathepsin L in CRC cell lines. Immunoblots of cell lysates from a panel of CRC cell lines demonstrated high variability in the total amount of legumain and cathepsin L, and also in the presence of the different mature forms. HCT116 and SW620 cells were particularly interesting as they show mutually exclusive high amount of the active (36 kDa) and inactive pro-form (56 kDa) of legumain, respectively. Uncut immunoblots (Fig. S2A).

Lysates from a panel of CRC cell lines were subjected to separation by PAGE and blotted onto PVDF membranes. By successive probing with polyclonal antibodies, the total amount and various mature forms of legumain (upper panel, Fig. 1 and Fig. S2A) and cathepsin L (middle panel, Fig. 1 and bottom panel, Fig. S2A) were visualized. Legumain appeared to be present in two molecular mass forms of approximately 56 and 36 kDa (arrows). The CRC cell lines displayed a wide range in the total amount of legumain, and also in relative amounts of the putative pro-form of 56 kDa and the active mature form of 36 kDa, which both were sensitive to down-regulation by a legumain specific siRNA (Fig. S1). Recombinant human pro-legumain (rhLeg, 5 ng) was used as control. Cathepsin L was present in most cell lines although the highest levels were seen in RKO, TC7 and HCT116. In the two latter, which harbor high amounts of mature legumain, the most dominant cathepsin L band corresponded to the 25 kDa heavy chain of the two-chain active form (arrow). In contrast, the RKO cell line showed simultaneous high expression of inactive (30 kDa, single chain) cathepsin L and a lower level of active legumain, suggesting that these cysteine proteases are subjected to mutual activation in colorectal cancer cell lines.

### The cell lines HCT116 and SW620 show divergent legumain activity

**Figure 2:**
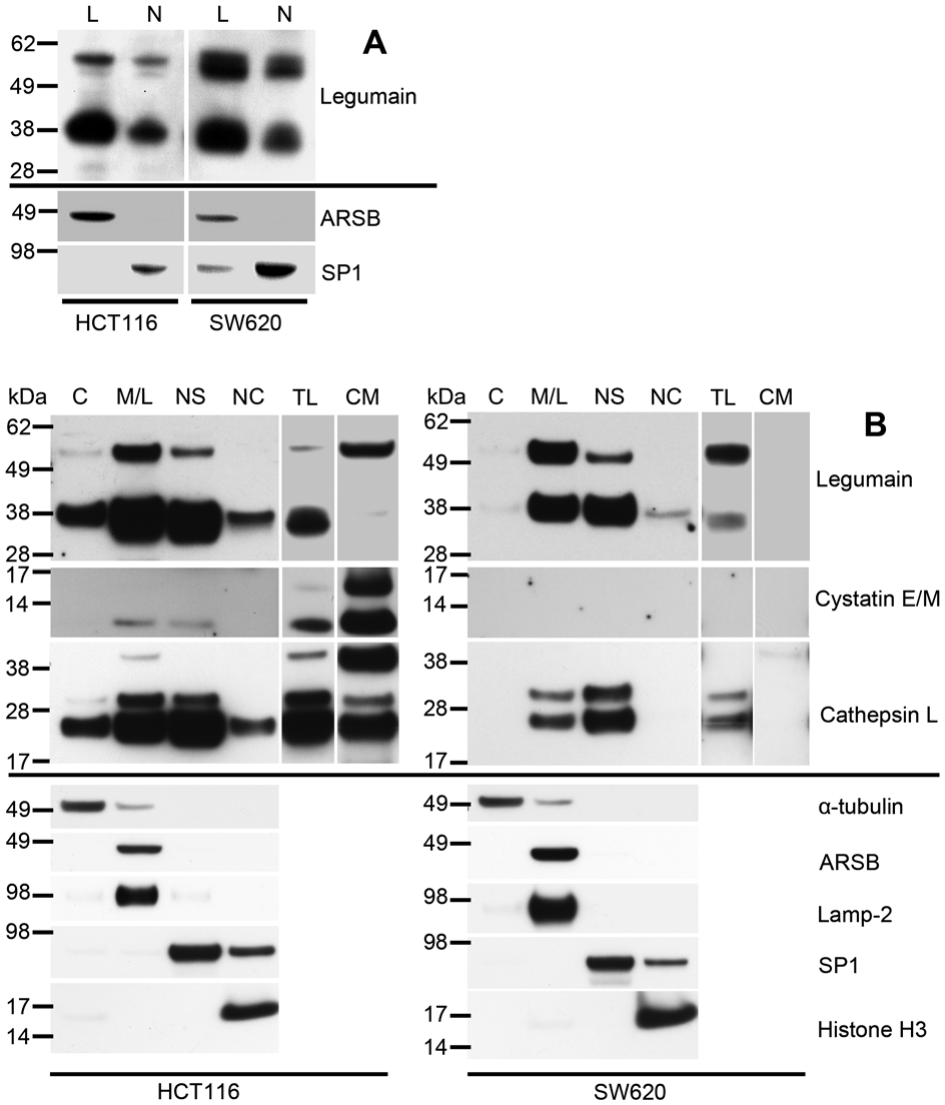
Legumain, cystatin E/M and cathepsin L expressions in subcellular fractions of HCT116 and SW620 cells. (A) Immunoblots of legumain in lysosomal (L) and nuclear (N) fractions enriched from HCT116 and SW620 cells using density gradient centrifugation. All lanes were loaded with 15 µg total protein from each fraction. Purity controls of the subcellular fractions were assessed by staining for ARSB (soluble lysosomal protein) and SP1 (nuclear transcription factor). (B) Immunoblots of legumain (top panels), cystatin E/M (second panels) and cathepsin L (third panels) in enriched subcellular compartments isolated from HCT116 and SW620 cells using a commercial kit: Cytosol (C), membranes/lysosomes (M/L), nuclear soluble (NS), nuclear chromatin bound (NC), total lysate (TL) and conditioned media (CM). All lanes were loaded with 15 µg total protein from each fraction, except conditioned media where proteins precipitated from 1 ml was loaded. Purity controls of the different subcellular fractions were assessed by staining for α-tubulin (cytosolic protein), ARSB (soluble lysosomal protein), lamp-2 (lysosome membrane-associated protein), SP1 (nuclear transcription factor) and histone H3 (nuclear chromatin bound protein). Uncut immunoblots of legumain and cathepsin L (Fig. S2C).

**Figure 3:**
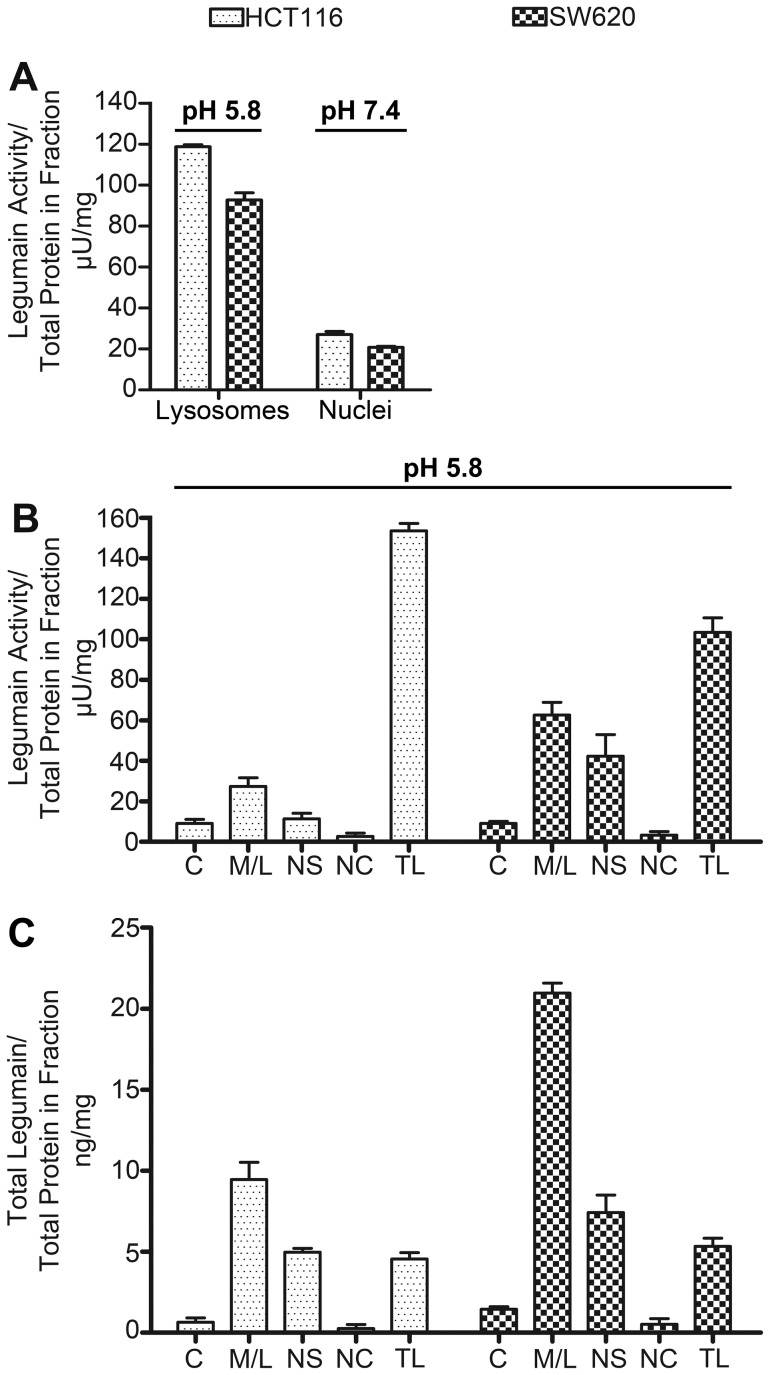
Proteolytic activity and quantity of legumain in subcellular fractions of HCT116 and SW620 cells. (A) Proteolytic activity of legumain determined by substrate cleavage (Z-Ala-Ala-Asn-NHMec) relative to total protein content of each subcellular compartment of HCT116 (dotted bars) and SW620 (chequered bars) cells after density gradient centrifugations. Lysosomal and nuclear fractions were prepared and analyzed at pH 5.8 and 7.4, respectively, demonstrating proteolytic activity of legumain in both lysosomal and nuclear fractions of both cell lines at both pH conditions, although highest in the lysosomal compartment assayed at pH 5.8. (B) Proteolytic activity of legumain measured at pH 5.8 in subcellular fractions prepared by a commercial kit was found to be highest in the M/L fractions, but was also clearly present in the NS fractions and observed with only minor activity in the C fractions of both cell lines. Extracellular legumain did not demonstrate any activity in either cell line (data not shown). (C) Total legumain amounts (pro- and active form) measured by ELISA in subcellular fractions (isolated using a commercial kit) and calculated relative to the total protein content in each fraction were also highest in the M/L fractions, yet clearly present in the NS fraction.

HCT116 cells predominantly displayed the mature 36 kDa form of legumain, whereas SW620 also showed substantial amounts of the 56 kDa pro-form (Fig. 1 and TL; Fig. 2B) [6]. Lysates from these two cell lines were further analyzed for their capability to cleave a legumain specific substrate. These activity measurements revealed a consistent correspondence between the observed intensity of the 36 kDa band and legumain activity in total lysates (TL; Fig. 3B). Furthermore, HCT116 and SW620 cells treated with a siRNA specific for legumain demonstrated a 70–90% decrease in legumain activity (data not shown). Having established evidence for the differences in amount of active legumain in HCT116 and SW620 it was of interest to determine whether this could be attributed to mutations in Asn323 located in exon 12, which has been claimed necessary for cleavage of the pro-enzyme into the active form [5], [6], [36]. However, sequencing showed no mutations in the *LGMN* nucleotide sequence corresponding to the cleavage recognition site in these cell lines (data not shown), and could thus not explain the observed difference in protease activity. Furthermore, cystatin E/M has been shown as the most potent endogenous inhibitor of legumain and expression of this protein was therefore investigated. Interestingly, the cellular and secreted levels of cystatin E/M were found to be quite different between the two cell lines. In HCT116, two forms (14 and 17 kDa) of cystatin E/M were found in conditioned media (CM; Fig. 2B), with more modest amount and mainly the 14 kDa form in the total cell lysate (TL). In contrast, SW620 expressed no detectable amounts of cystatin E/M neither secreted nor intracellular.

### Active legumain is localized to the endo-lysosomal and nuclear compartments

Legumain is usually considered a protein targeted to and residing in endo-lysosomes, and it has also been reported to execute important cellular functions inside this specialized compartment [22], while its distribution and characteristics in other subcellular compartments has not been elucidated. To further investigate this we used two methods to isolate and enrich proteins from subcellular compartments, one being based on density gradient centrifugation in sucrose buffer and the other being a commercially available subcellular isolation kit. To examine the distribution of legumain, immunoblotting was performed on 15 µg total protein lysate from each enriched subcellular fraction, in addition to total cell lysate and corresponding conditioned growth medium (Fig. 2). This also enabled for purity control of the fractions using various proteins of presumed limited distribution, showing high enrichment of each compartment and with almost no detectable cross contamination (Fig. 2, compartment-specific markers displayed below black lines). In both subcellular enrichment methods the 36 kDa active legumain was present in substantial amounts in the lysosomal (L) and membrane/lysosomal (M/L) fractions, as well as in the nuclear (N) and nuclear soluble (NS) fractions in both both cell lines. It was again perceived that overall HCT116 compared to SW620 cells contained a lower level of the 56 kDa pro-form in all subcellular fractions, confirming the observations made from total cell lysates (Fig. 1 and 2), while the 56 kDa prolegumain was also observed in the nuclear fractions in both cell lines. HCT116 cells also displayed smaller amounts of 36 kDa legumain in the cytosolic (C) and nuclear chromatin bound (NC) fractions (Fig. 2B and Fig. S2C, left middle panels). The 56 kDa pro-form of legumain was found to be secreted and detected in conditioned media (CM; Fig. 2B), but only from HCT116. Of particular interest was the clear presence of the 36 kDa active legumain in the nuclear fractions from both cell lines, in addition to the anticipated presence in the lysosomal fractions. Additionally, subcellular enrichment was performed using a second commercially available subcellular isolation kit (Qiagen), also demonstrating the nuclear localization of mature 36 kDa legumain (Fig. S2B). Regarding the endogenously expressed inhibitor cystatin E/M, only trace amounts of the 14 kDa form was observed in both the M/L and NS fraction of HCT116. Finally, subcellular distribution of cathepsin L was also evaluated and this protease was found to be much less prominent in SW620 compared to HCT116 cells (Fig. 2B and Fig. S2C, lower panels), as was also apparent in total cell lysates (Fig. 1 and 2B). In HCT116 the presumed 25 kDa active two-chain form of cathepsin L [37] was clearly present in the M/L and NS fractions as well as in TL and CM, but also to some extent in the C and NC fractions. Although present in active form in the CM, this fraction also showed the pro-form of cathepsin L viewed by a strong band of approximately 38 kDa. The cathepsin L single-chain form of 30 kDa was mainly present in the M/L and NS fractions of both cell lines. In SW620, weaker bands of cathepsin L were observed only in the M/L and NS fractions as well as in TL, and also with a faint presence of the pro-form in CM.

The various subcellular fractions from HCT116 and SW620 cells were subsequently analyzed for their capability to proteolytically cleave a legumain specific peptide substrate relative to the total amount of proteins present in each measured fraction. In lysosomal and nuclear fractions separated by sucrose density gradients, the activity was measured at both pH 5.8 and 7.4, respectively, to mimic physiological conditions (Fig. 3A). In conjunction with previous studies, the lysosomal fractions demonstrated high proteolytic legumain activity, but more unanticipated the nuclear fractions of both cell lines measured at neutral pH also showed considerable legumain activity. In the subcellular fractions isolated using the commercial kit, proteolytic activity of legumain measured at pH 5.8 was also highest in the M/L (Fig. 3B) fraction, however, in correspondence with previous results, substantial legumain activity was found in the nuclear fractions of both cell lines, in particular the soluble fractions (NS). Furthermore, legumain expression in the subcellular fractions obtained by using the commercial kit was assessed using sandwich ELISA capable of detecting total legumain (i.e. both pro- and active forms) (Fig. 3C). In all fractions detectable amounts of legumain (relative to total protein content in each fraction) were observed, while the highest levels were observed in the M/L and NS fractions, as also seen on the immunoblots (Fig. 2B). An unanticipated result was the quantity and activity of legumain in the subcellular fractions from 10*10^6^ SW620 cells which were measured to be approximately twice as high as in fractions made from 5*10^6^ HCT116 cells, although all lanes were loaded with 15 µg protein. However, in total lysates the entire amount of legumain (pro- and mature-form) was nearly equal, yet HCT116 cells had higher overall legumain activity in the total cell lysates which corresponds to the observed amount of active legumain in the immunoblots (Fig. 2B).

### *In situ* distribution of legumain expression

**Figure 4:**
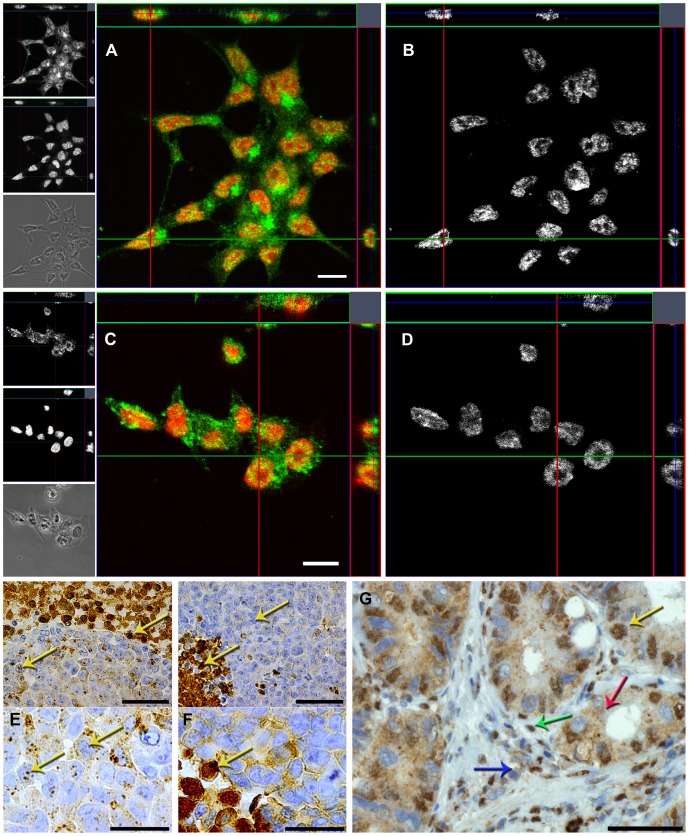
Subcellular localization of legumain in HCT116 and SW620 cells, subcutaneous xenografts, and human CRC tumor tissue. (A to D) Immunofluorescence staining of legumain (green and top left panels) and nuclei (red and middle left panels) in HCT116 (A) and SW620 (C) cells cultured on glass slides and visualized as orthogonal slices of z-stacks by confocal laser scanning microscopy. By using image arithmetics with the binarized capture of corresponding DRAQ5™-positive nuclei as a mask, nuclear legumain representative signals were extracted from all optical sections and visualized in grayscale as orthogonal slices for HCT116 (B) and SW620 (D). Scale bars represent 10 µm. Specificity of immunofluorescence signals was verified by incubation with secondary antibodies only, yielding no signal (Fig. S3A and S3B). (E and F) When grown as subcutaneous xenografts in mice, immunohistochemical staining of legumain in HCT116 (E) cells demonstrated a much more granulated staining pattern than in SW620 (F) cells. However, both cell lines exhibited areas of strong legumain expression and also in the nuclei (yellow arrows). Scale bars represent 50 (top panels) and 25 (bottom panels) µm. H/E stain (Fig. S3C and S3D). Goat-IgG isotype control showed low staining (Fig. S3E and S3F). (G) Immunohistochemical staining of legumain in paraffin-embedded section from a representative CRC tumor biopsy showing nuclear staining of legumain in some, but not all, epithelial cells (i.e. carcinoma cells; yellow arrow) and stromal cells (green arrow). Epithelial cells also exhibited marked granulated staining in the cytoplasm (red arrow), whereas stromal cells showed much weaker staining outside the nucleus (blue arrow). Scale bar represent 50 µm. Goat-IgG isotype control showed no staining (Fig. S3G).

**Figure 5:**
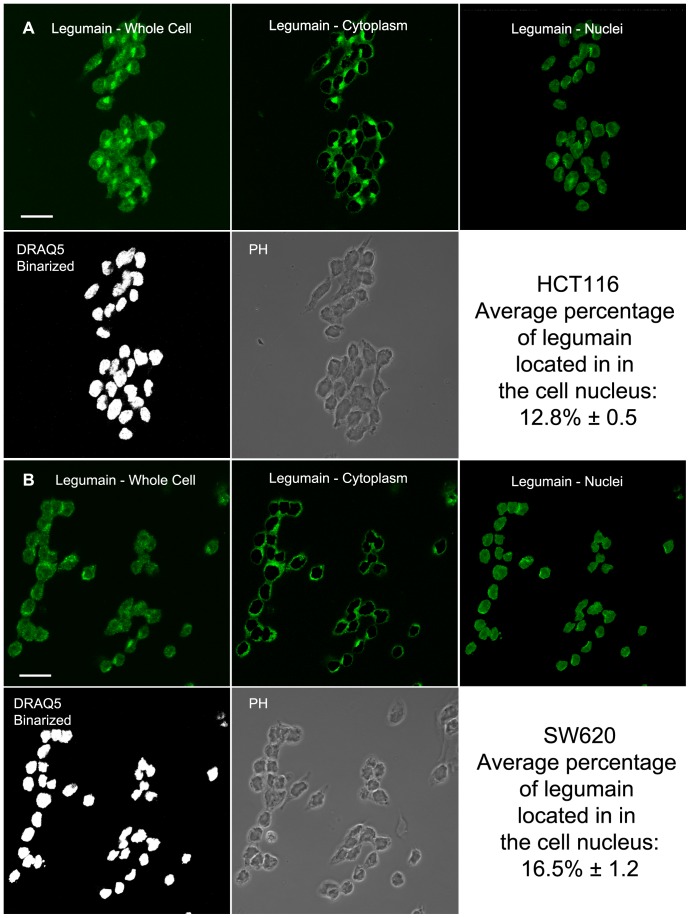
Percentage of expressed legumain located in the nucleus. (A and B) Representative pictures of HCT116 (A) and SW620 (B) cells by one optical slice from one of five independent z-stacks each containing 30–40 immunofluorescently labeled cells using legumain-specific antibodies (green), with DRAQ5™ counter-stained nuclei (binarized; white). By means of a semi-automated procedure in Image J the captures of nuclei (binarized) was used as a mask to separate the nuclear (top right) from the cytoplasmic (top middle) signal components of the total (top left) signal representing expression of legumain. The total signal from legumain fluorescence in each optical slice was summarized from all five z-stacks enabling for the estimation of the expressed amount of legumain in the nuclear compartment. Statistical errors in the calculations are reported as standard error of the mean of the five independent z-stacks. Scale bar represents 20 µm.

To examine legumain expression *in situ*, HCT116 and SW620 cells were cultured on glass slides, immunofluorescently stained and visualized using confocal microscopy (Fig. 4A–4D and Fig. S3A–S3B). Legumain was mainly distributed in the perinuclear region, possibly suggesting majorly localization to the *trans*-Golgi network (TGN) and endo-lysosomes, and most clearly visible in the HCT116 cells (Fig. 4A), whereas in the SW620 cells legumain-containing vesicles appeared more distributed (Fig. 4C). Both HCT116 and SW620 also displayed legumain located in the nuclei of the cells. By visualizing only the nuclear localized legumain in all three orthogonal planes of the cells, legumain was observed to distribute around spherical structures, possibly nucleoli, inside the nuclei (Fig. 4B and 4D). Using image arithmetics on optical slices from five independent z-stacks, each containing 30–40 immunofluorescently labeled cells, the average percentage of intracellular legumain localized in the nucleus of a cell was estimated to 12.8% in HCT116 and 16.5% in SW620 (Fig. 5). Additional analysis of the two cell lines grown as subcutaneous xenografts in mice revealed comparable results to immunofluorescence labeling when immunohistochemically stained for legumain (Fig. 4E and 4F, and Fig. S3C–S3F), with HCT116 showing intense granulated staining whereas SW620 demonstrated a much more diffuse staining pattern. Furthermore, in xenografts from both cell lines legumain exhibited heterogenous expression, with intensely stained areas, possibly necrotic tissue. Nuclear localization was most visible in cells with overall high legumain expression, but spots of legumain staining were also detected inside nuclei in the weaker stained cells (yellow arrows). Immunohistochemical analysis of paraffin embedded tumor tissue from CRC patients, also revealed heterogeneous expression of legumain which was apparent both in the tumor cells and the surrounding stromal cells (Fig. 4G and Fig. S3G). Most strikingly are the intensely stained nuclei present in about 50% of the tumor cells and 30% of the stromal cells (yellow and green arrow, respectively), whereas other adjacent cells demonstrated no nuclear staining. Furthermore, the overall staining pattern showed elevated cytoplasmic presence of legumain in all apparent carcinoma cells compared to the stromal cells (red and blue arrow, respectively).

### *In situ* distribution of legumain activity

**Figure 6:**
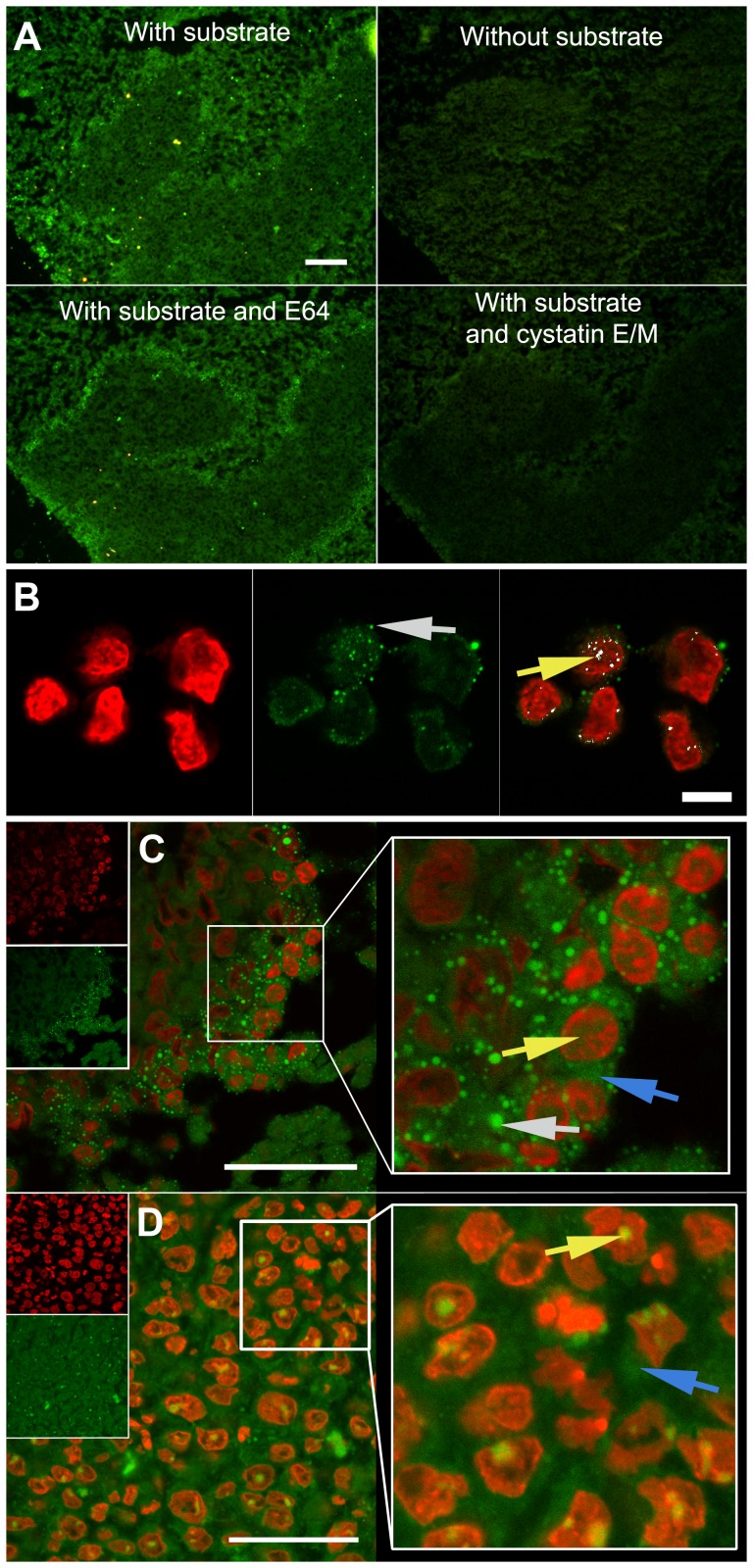
*In situ* legumain activity in cultured cells and subcutaneous xenografts. (A) *In situ* proteolytic activity (green) captured by fluorescence microscopy imaging of adjacent cryosections from a HCT116 subcutaneous xenograft incubated with (top left) and without (top right) legumain substrate, legumain substrate and E64 (lower left) or legumain substrate and recombinant cystatin E/M (lower right), demonstrating the specificity of the synthetic peptide Suc-Ala-Ala-Asn-NHNapOME utilized as legumain substrate. All pictures were taken using true colors, after the same incubation time and with identical microscope and camera settings. Scale bar represents 100 µm. Subcutaneous xenografts with SW620 cells (Fig. S3J). (B) Subcellular localization of active legumain (green) in HCT116 cells (made from cryosections after mounting in OCT-medium) with nuclei stained by DAPI (red) and analyzed by confocal laser scanning microscopy. This showed granulated activity inside (yellow arrow) and outside (gray arrow) of the nucleus. Localization in the nucleus was confirmed by co-localization (white) of legumain activity and the nuclear counter-stain (right panel). Scale bar represents 10 µm. HCT116 cells incubated without substrate, or with substrate and cystatin E/M, showed no signals (Fig. S3H and I, respectively). (C and D) Legumain activity (green) in cryosections from subcutaneous xenografts with nuclei stained with DAPI (red) and analyzed by confocal laser scanning microscopy. Subcutaneous xenograft from HCT116 cells (C) showed similar results as in cultured cells with intense granulated activity (gray arrow) although less distinct activity in the cytoplasm (blue arrow) and within the nucleus (yellow arrow) was also observed. However, in the subcutaneous xenograft of SW620 cells (D) majorly diffuse legumain activity was observed in the cell cytoplasm (blue arrow), while in the nucleus this was more concentrated (yellow arrow). Scale bars represent 50 µm.

Having established nuclear expression of legumain *in situ,* it was of interest to see whether proteolytic activity also could be verified in this compartment of intact cells. Performance and specificity of a legumain specific substrate was verified on non-fixed cryosections from xenografts by incubation with or without the substrate (Fig. 6A, upper pictures and Fig. S3J) demonstrating the presence of active legumain, while addition of the E64 cathepsin inhibitor did not appear to affect the cleavage of the substrate (Fig. 6A, lower left picture and Fig. S3J). In contrast, incubation with recombinant cystatin E/M completely abolished detectable legumain activity (Fig. 6A, lower right picture and Fig. S3J), altogether confirming the legumain specificity of the chosen substrate at the established conditions. This substrate was further used to analyze *in situ* legumain activity on cryosections from HCT116 cells (Fig. 6B) and xenografts from both cell lines (Fig. 6C and 6D). In HCT116 cells, legumain activity in the cytoplasm appeared diffuse, but also with more bright and distinct spots (Fig. 6B, middle, gray arrow). In addition, activity was observed as distinct spots (right, yellow arrow) in the cell nucleus viewed by co-localization (white). On cryosections made from xenografts established from HCT116 cells, legumain activity in the cell nucleus (Fig. 6C, yellow arrow) was less strong and distinct, whereas small intense vesicles could be observed in the cytoplasm (gray arrow) together with more diffuse (blue arrow) activity. Parallel analysis of xenografts made from SW620 cells showed less distinct activity in the cytoplasm (Fig. 6D, blue arrow), but was quite focused in the nucleus (yellow arrow). Altogether, the observations on legumain expression and activity *in situ* confirmed the findings by immunoblotting and activity measurements of subcellular fractions, and further revealed differences between HCT116 and SW620 cells in legumain distribution. Nonetheless, both cell lines showed presence of active legumain in the nucleus.

### Histone H3.1 is proteolytically cleaved by legumain

**Figure 7:**
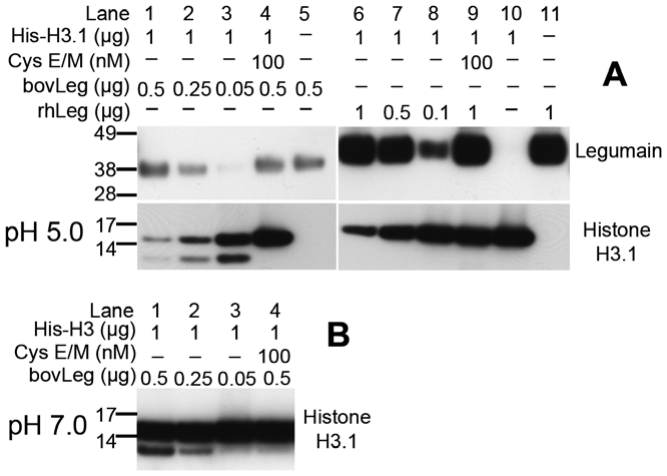
Cleavage of histone H3.1 by active legumain. (A) Immunoblots showing the cleavage of intact (lane 10) recombinant human histone H3.1 in a dose dependent manner by purified mature 36 kDa bovine legumain (bovLeg, lane 1–3) and auto-activated intermediate form (46 kDa) of recombinant human legumain (rhLeg, lane 6–8). The addition of recombinant human cystatin E/M (lane 4 and 8) efficiently blocked legumain activity and resulted in almost complete rescue of histone H3.1 from proteolytic cleavage. Uncut immunoblots (Fig. S2D). (B) Immunoblot of histone H3.1 showing the dose-dependent production of a 12 kDa cleavage product after incubation of recombinant histone H3.1 with fully mature 36 kDa bovine legumain in a buffer with pH 7.0 (lane 1–3). Addition of recombinant human cystatin E/M efficiently blocked legumain activity and resulted in virtually no formation of the 12 kDa cleavage product (lane 4).

Given the presence of active legumain in the nucleus and in line with a previous report on cathepsin L [38] it was of interest to investigate whether legumain was able to cleave the nuclear protein histone H3. By the use of auto-activated human recombinant legumain produced in mammalian cells, it was demonstrated that incubation at pH 5.0 resulted in a dose dependent proteolytic cleavage of recombinant histone H3.1 (Fig. 7A and Fig. S2D, lanes 6–8), which was nearly abolished in presence of cystatin E/M (Fig. 7A and Fig. S2D, lane 9). The auto-activation of legumain only produced the intermediate 46 kDa form of the protease, hence not the endogenously observed and presumably fully matured 36 kDa form. We therefore repeated the experiment using fully matured legumain purified from bovine kidney, and detected an even higher proteolytic activity resulting in near complete cleavage of histone H3.1, including the appearance of an approximately 12 kDa cleavage product which apparently also was a substrate for further legumain processing (Fig. 7A and Fig. S2D, lanes 1–3). Addition of cystatin E/M completely blocked cleavage of histone H3.1 by bovine legumain (Fig. 7A and Fig. S2D, lane 4). The experiment was also conducted under pH conditions adjusted to 7.0, which resulted in appearance of the 12 kDa histone H3.1 cleavage product in a dose dependent manner using the fully matured 36 kDa legumain, which was blocked by cystatin E/M (Fig. 7B, lane 1–4). The proteolytic cleavage rate for fully matured legumain was apparently much slower at neutral than at acidic pH, as most of the intact histone was observed. The 46 kDa intermediate active form of legumain did not result in any cleavage products at neutral pH (not shown).

## Discussion

In consistence with previous reports [10], [39], legumain and cathepsin L were found ubiquitously expressed in a panel of CRC cell lines. However, clear variations in protease amounts and ratios between the various maturation forms of each protease were identified. Both legumain and cathepsin L require a multistep activation process to reach their mature forms. These maturation processes are not completely elucidated, but both autocatalytic activity and an interplay between the various cysteine proteases have been suggested [6], [7], [40]. However, based on the presented immunoblots no statistically significant co-variation in the expression levels of the analyzed proteases was observed, nor was it possible to decipher any consistency in the total amount of the presumed active forms. This could be ascribed to the complexity of the maturation process in which these two cysteine proteases represent only some of all proteases involved. Furthermore, influences caused by variations in experimental conditions like pH and cell density could add to the complexity in the interpretation of the results.

The difference in relative amounts of the 56 and 36 kDa legumain forms, representing the zymogen and the mature active protease, respectively [4], [6], [13], was most prominent between the cell lines HCT116 and SW620. Several explanations for the observed differences in legumain processing, and thus proteolytic activity, could be postulated. Initially, exon 12 in the *LGMN* gene, harboring a presumed crucial target for processing to the active 36 kDa form, was sequenced without revealing any mutations that could explain the lower level of mature legumain in SW620. Secondly, insufficient buffering capacity of the cultivating medium could possibly influence intracellular functions. However, measurements of pH prior to cell harvesting did not reveal differences in the experimental conditions between the cultures of both cell lines (data not shown). Furthermore, the legumain inhibitor cystatin E/M was found in HCT116, but not in the SW620 cell line. In line with the original reports [41], [42] we also found two molecular mass forms of cystatin E/M. The 17 kDa was mainly secreted whereas the 14 kDa form was both secreted and dominant in lysates from HCT116 cells. Previously, we have reported an inverse correlation between secreted 17 kDa cystatin E/M and active legumain in melanoma cells [13]. Cystatin E/M is supposed to inhibit proteolytic activity of mature legumain, and although part of the maturation process is autocatalytic there are no indications that the inhibitor *per se* restrains processing towards active protease [40], possibly as a consequence of physical separation of the two proteins during the maturation process [43]. Therefore, discrepancy in cystatin E/M expression can probably not explain the observed differences in mature 36 kDa legumain between the two colon cancer cell lines. Finally, addition of ammonium chloride in the growth media to increase lysosomal pH and inhibit legumain maturation [36], did remove all detectable 36 kDa legumain (data not shown). Thus, lysosomal maturation seems to be primarily responsible for the appearance of the active form, and speculations can be made to whether aberrant lysosomal transport or function could explain low activity in SW620. This hypothesis is in concurrence with our observations from immunohistochemistry and *in situ* activity measurements on subcutaneous xenografts showing a more granulated localization of legumain expression and activity in HCT116 than in SW620 cells. Furthermore, immunofluorescence imaging demonstrated that legumain is more distributed throughout the entire cell in SW620 compared to the main presumed TGN and lysosomal location in HCT116 cells.

The dominant theory for intracellular transport of legumain is through the TGN and further into lysosomes via late endosomes, where trimming by autocatalysis and other proteases takes place [7], [40]. Anyhow, cysteine proteases are also known to be subject to alternative trafficking in the cells [44]. In line with other studies [45], the highest amounts of legumain were observed in the intra-organelle membrane fraction (Golgi, endosomes, lysosomes), but substantial amounts of legumain were surprisingly also found in the cell nucleus. The amount of legumain relative to the total protein content in each compartment differs from the amount compared to total cellular protein, possibly explaining why more active legumain was observed in the subcellular compartments of the SW620 cell line than in the total cell lysate. Another explanation could be that some of the pro-form is cleaved during experimentation giving rise to the mature active form. The ratio between pro- and active form in the lysosomal fractions of HCT116 is in concurrence with the observed ratio in total cell lysate, and suggests rapid processing to the mature 36 kDa form within the lysosomes/membrane structures. Although higher amounts of active legumain were observed in the M/L fraction of SW620 cells than in the total lysate, the substantial presence of pro-legumain in the M/L fraction suggests that even though legumain is transported to the endo-lysosomes, its processing is not as efficient in SW620 as in HCT116 cells. In line with a previous report [6], only the pro-form was detected extracellularly, and interestingly only from the HCT116 cell line, although SW620 demonstrated high levels of this form intracellularly. This was also reflected by the total absence of legumain activity in conditioned media (data not shown), and suggests that the protease either does not mature or is unstable in its active form under the culturing conditions used. Legumain activity has, however, recently been reported in conditioned media from other cell lines [19], suggesting that certain growth conditions (e.g. hypoxia often encountered in tumors) would be more favorable towards protease maturation, and furthermore, that the active form of legumain may be stabilized by certain co-factors [5]. This newly proposed theory of stabilizing co-factors may also explain how legumain could be active in the nuclear compartment which presumably does not have the optimal pH, which was observed for the nuclear fractions showing considerable proteolytic activity at pH 7.4. One candidate could be histones, also demonstrated as a potential substrate in this study, alone or in complex with DNA, previously revealed to act as a template for cysteine cathepsin interaction with endogenous protease inhibitors that serve as substrates [46].

Legumain expression and activity was observed in the nucleus of both CRC cell lines analyzed in this study. This novel finding was first demonstrated by immunoblotting, ELISA and proteolytic activity measurements, and further confirmed by immunofluorescence and detection of enzymatic activity *in situ* on both intact cells and xenografts in addition to immunohistochemistry on tissue from xenografts and on human CRC tumor tissue. Yet, the cellular route taken by legumain to reach the nucleus appears enigmatic, with both the pro- and active forms observed in this subcellular compartment. Analysis of the legumain amino acid sequence with a nuclear localization signal (NLS) prediction algorithm [47] returned two potential NLSs in the C-terminal region with a score suggesting strong, but not exclusive, localization to the nucleus (Fig. S4). Interestingly, the predicted mono- and bipartite NLS signal are located on each side of the predicted maturation site at N323, which could be of importance in favoring nuclear import before or after maturation of legumain. In support of the hypothesis that maturation cleavage takes place before nuclear import, was our finding of low expression of prolegumain in the nuclear fractions of HCT116 and SW620 cells, but this is a topic that needs further exploration. For future studies, although not within the scope of this report, it seems vital to explore the proteolytic network of either cell line in a more comprehensive approach since also amounts and differential localization patterns of e.g. cysteine cathepsins may add to the extent of prolegumain processing including unmasking of nuclear targeting sequences.

Although acidic pH has been reported as optimal for activity and stability of legumain [4]–[6], proteolytic activity of the nuclear fractions at neutral pH was observed. Furthermore, cleavage of histone H3.1 at pH 7.0, which is close to presumed physiological pH in the nucleus, was also seen. The activity was less prominent than at pH 5.0, but as previously mentioned, certain co-factors may stabilize the mature form and promote the proteolytic activity at neutral pH [5]. In addition, proteolytic activity at neutral pH has been reported for cathepsins [48] and legumain in *Blastocystis* [49]. In line with previous reports, the *in situ* activity measurements on cells and tissue demonstrate that the most prominent legumain activity was observed as granulated spots in the cytoplasm of HCT116 cells, probably representing the endo-lysosomes. These vesicles have high levels of legumain and optimal pH for activity, and are thus regarded as the canonical location for legumain activity. As the percentage of nuclear localized legumain was demonstrated to be only minor and not exceeded 17% of the total detectable legumain, such vesicles might therefore in previous studies have masked less prominent locations like the nucleus. For certain biological processes high substrate cleavage rate is essential, but for others the processing of a given protein even at a slower rate may be very important.

To our knowledge, legumain expression and activity have not previously been examined in the cell nucleus, while several other cysteine proteases like caspase [50], separase [51] and variants of cathepsins [38], [52]–[54] are reported to execute proteolytic activity in this subcellular compartment. Furthermore, DNA has been demonstrated to act as a template for cathepsins and their inhibitors, and regulate the proteolytic activity [46]. In the present study nuclear localization was confirmed for cathepsin L in both HCT116 and SW620 cells, but less prominent in the SW620 cells, although nuclear active legumain was found in both cell lines. We have recently reported the nuclear localization of the endogenous legumain inhibitor cystatin E/M [55]. Nuclear forms of cathepsin L has previously been reported to be involved in proteolytic processing of transcription factors [52], [53] and regulation of histone H3 during mouse embryonic stem cell differentiation [38]. However, evidence for nuclear localized legumain and the possibility of histone H3.1 as a potential legumain substrate has not previously been described. The cysteine proteases are known to interplay during the protease maturation process, and legumain has been shown to be involved in processing of cathepsin L [56]. Thus, speculations could be made towards reciprocal involvement, or redundancy, of legumain and cathepsin L forms in their biological functions in the cell nucleus. Interestingly, legumain was recently reported to proteolytically process the nuclear protein SET [57] and TDP-43 [58], but the exact subcellular location of this process remained elusive.

In recent years, research on legumain has gained momentum, reflecting the significance in cancer progression and potential as a therapeutic target. However, knowledge about subcellular localization, requirements for activation and proteolytic activity remained largely unexplored as of now. This study demonstrated that legumain maturation is not identical in all CRC cells, and this is probably due to factors other than alterations of the amino acid chain of the protein *per se,* possibly intracellular trafficking or absent cleavage by other proteases. Most importantly, the study is the first to address legumain expression and proteolytic activity in the nucleus of CRC cells. Histone H3.1 was demonstrated to be a potential legumain substrate, but the *in vivo* functional implications of legumain activity in the nucleus are yet to be explored. The presented data enhance our knowledge on legumain biology, but further studies are warranted to elucidate the contribution of legumain processing and localization in cancer development and progression.

## Supporting Information

Figure S1 **Down-regulation of legumain with siRNA demonstrated specificity of the utilized antibody.** Sub-confluent cultures were transfected with 10 nM siRNA specific for legumain (Ambion) or Select Negative Control 2 (Ambion) using Lipofectamine (Invitrogen) and Opti-MEM I (Invitrogen). After 24 h the growth medium was changed and cells grown for an additional 48 h before harvesting. Immunoblot stained with the legumain antibody demonstrated that both the 56 pro- and 36 kDa mature form of legumain are down-regulated in siRNA-treated cells. α-tubulin was used as loading control.
(TIF)

Figure S2 **Uncut immunoblots from** **figure 1** **,** **2** **and** **7** **, and additional subcellular enrichment.** (A) Uncut immunoblots of legumain (upper panel) and cathepsin L (lower panel) in Fig. 1, respectively. The bands detected around 98 kDa forms in TC7 and SW620 are thought to be a dimeric form of the 56 kDa prolegumain. (B) Subcellular enrichment using a kit from Qiagen demonstrating nuclear localized 36 kDa legumain in HCT116 and SW620 cells. Cytosol (C), membranes/lysosomes (M/L) and nuclei (N). Purity controls of the subcellular fractions were assessed by staining for the proteins α-tubulin (cytosolic), lamp-2 (lysosomal) and Lamin-B (nuclear). (C) Uncut immunoblots of legumain from subcellular fractions in Fig. 2A (upper panels), and from subcellular fractions in Fig. 2B legumain (middle panels) and cathepsin L (lower panels). (D) Uncut immunoblots of legumain (upper panels) and histone H3.1 (lower panels) in Fig. 7A. Legumain was immunostained on identical blots after stripping off anti-histone H3.1 and the respective secondary antibody, thus some residual signal of intact histone H3.1 at 17 kDa remained.
(TIF)

Figure S3 **Negative controls for legumain immunofluorescence, immunohistochemistry and** ***in situ*** **activity, and H/E staining of subcutaneous xenografts.** (A and B) HCT116 and SW620 cells, respectively, incubated without primary legumain antibody and stained with Alexa488 labeled rabbit-anti-goat antibody and nuclei stained with DRAQ5™. Scale bars represent 10 µm. (C and D) Hematoxilin/Eosin staining of subcutaneus xenografts from HCT116 and SW620 cells, respectively. Scale bars represent 50 µm. (E and F) Goat-IgG isotype control staining of subcutaneus xenografts from HCT116 and SW620 cells, respectively. A very faint, diffuse background staining was observed in tumor cells, while this was more pronounced in certain areas, possibly necrotic tissue. Scale bar represents 50 µm. (G) Goat-IgG isotype control staining of human colorectal tumor tissue. Scale bar represents 50 µm. (H and I) HCT116 cells incubated with buffers for *in situ* legumain activity without presence of the cleavable substrate (H) and with substrate and 100 nM recombinant cystatin E/M (I). Scale bars represent 10 µm. (J) Subcutaneous xenografts from SW620 cells incubated with and without substrate, and with substrate and E64 or cystatin E/M. Scale bar represents 200 µm.
(TIF)

Figure S4 **Predicted NLS in legumain.** The FASTA sequence of legumain (Q99538) was analyzed for potential NLS signals using NLS-mapper. The return score for monopartite (aa284–293) and bipartite signals (aa313–342) of 6 and 5.1, respectively, indicated a moderately strong, but not exclusive, nuclear localization signal in the protein.
(TIF)
