# Correction: Nuclear Legumain Activity in Colorectal Cancer

**DOI:** 10.1371/annotation/05c95441-890f-4707-a1bc-c4d386561191

**Published:** 2013-09-19

**Authors:** Mads H. Haugen, Harald T. Johansen, Solveig J. Pettersen, Rigmor Solberg, Klaudia Brix, Kjersti Flatmark, Gunhild M. Maelandsmo

A following funder was erroneously omitted:Jacobs University Bremen [2140/90140].

The correct financial disclosure is: This work has been generously supported by grants from South-East regional health authorities [HSØ, #2011142], The Norwegian Research Council under the Functional Genomics Program [FUGE, #158954/S10], Søren and Jeanette Bothners legacy and the Norwegian Cancer Society [#PR-2006-0272], Jacobs University Bremen [2140/90140], Deutscher Akademischer Austausch Dienst (DAAD) [A/11/95548], The University of Oslo, Anders Jahres foundation for the Promotion of Science, Astrid and Birger Torsteds Foundation, and the Nansen Foundation. The funders had no role in study design, data collection and analysis, decision to publish, or preparation of the manuscript. 

