# Supplementary figures and images for: Nuclear Legumain Activity in Colorectal Cancer
Source: PLoS One. 2013 Jan 10;8(1):e52980. doi: 10.1371/journal.pone.0052980 (PMC3542341; doi:10.1371/journal.pone.0052980)

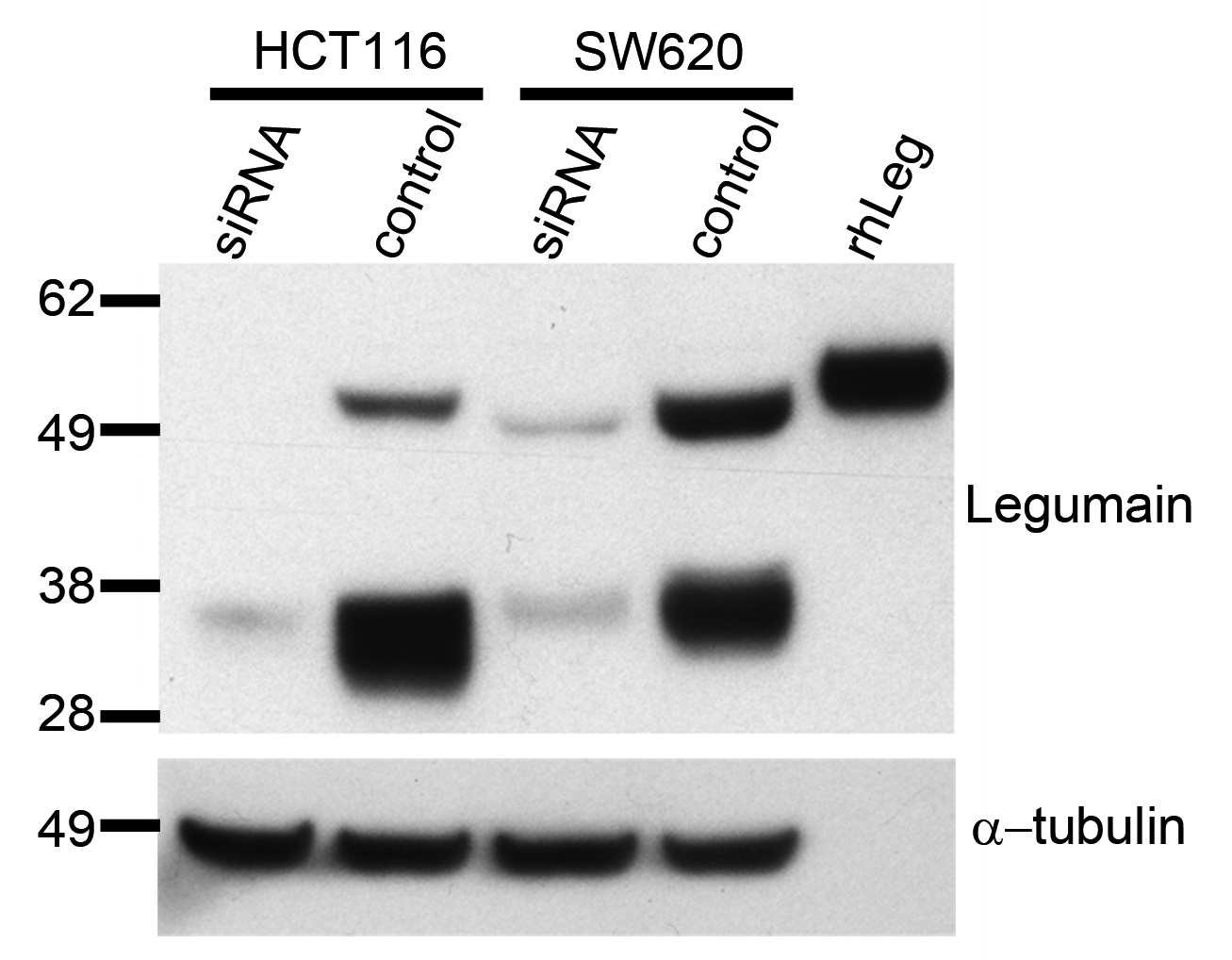

Supplement: Figure S1 — Down-regulation of legumain with siRNA demonstrated specificity of the utilized antibody. Sub-confluent cultures were transfected with 10 nM siRNA specific for legumain (Ambion) or Select Negative Control 2 (Ambion) using Lipofectamine (Invitrogen) and Opti-MEM I (Invitrogen). After 24 h the growth medium was changed and cells grown for an additional 48 h before harvesting. Immunoblot stained with the legumain antibody demonstrated that both the 56 pro- and 36 kDa mature form of legumain are down-regulated in siRNA-treated cells. α-tubulin was used as loading control. (TIF) [file pone.0052980.s001.tif]

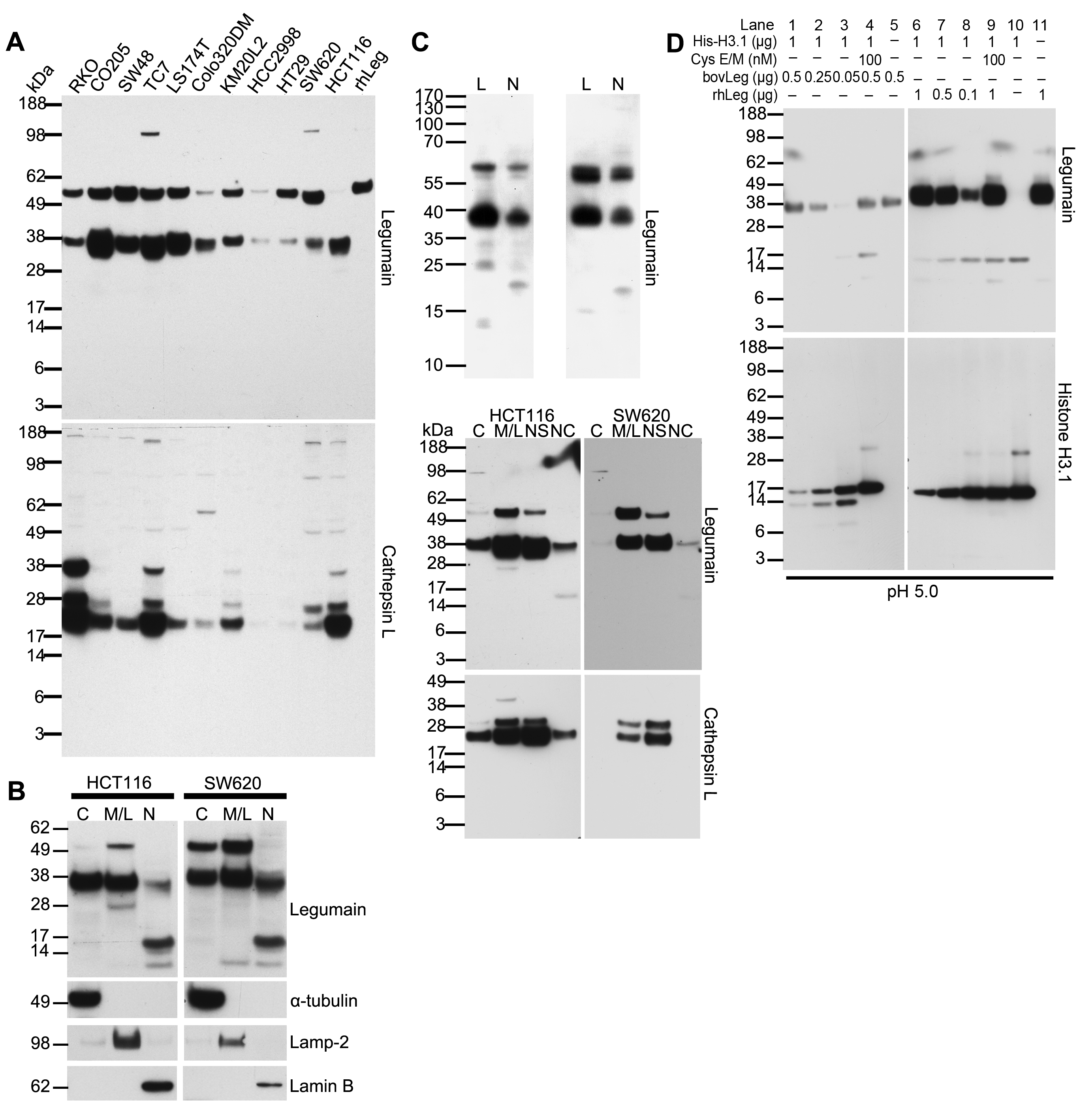

Supplement: Figure S2 — Uncut immunoblots from figure 1 , 2 and 7 , and additional subcellular enrichment. (A) Uncut immunoblots of legumain (upper panel) and cathepsin L (lower panel) in Fig. 1, respectively. The bands detected around 98 kDa forms in TC7 and SW620 are thought to be a dimeric form of the 56 kDa prolegumain. (B) Subcellular enrichment using a kit from Qiagen demonstrating nuclear localized 36 kDa legumain in HCT116 and SW620 cells. Cytosol (C), membranes/lysosomes (M/L) and nuclei (N). Purity controls of the subcellular fractions were assessed by staining for the proteins α-tubulin (cytosolic), lamp-2 (lysosomal) and Lamin-B (nuclear). (C) Uncut immunoblots of legumain from subcellular fractions in Fig. 2A (upper panels), and from subcellular fractions in Fig. 2B legumain (middle panels) and cathepsin L (lower panels). (D) Uncut immunoblots of legumain (upper panels) and histone H3.1 (lower panels) in Fig. 7A. Legumain was immunostained on identical blots after stripping off anti-histone H3.1 and the respective secondary antibody, thus some residual signal of intact histone H3.1 at 17 kDa remained. (TIF) [file pone.0052980.s002.tif]

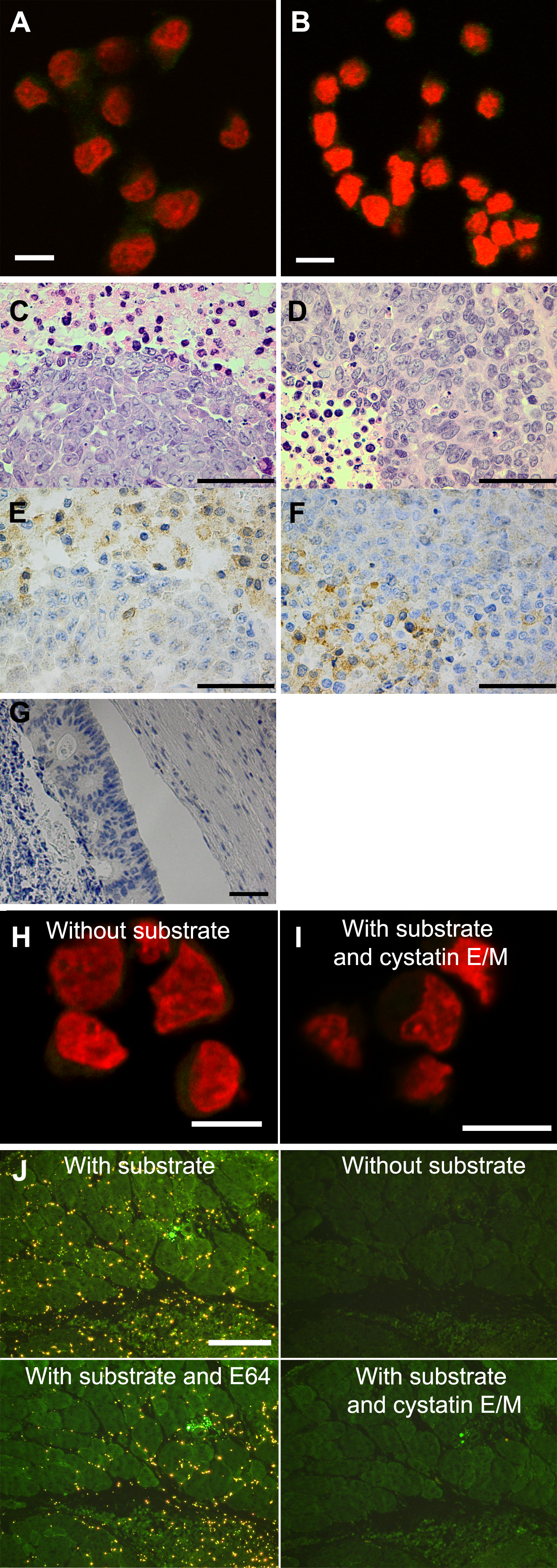

Supplement: Figure S3 — Negative controls for legumain immunofluorescence, immunohistochemistry and in situ activity, and H/E staining of subcutaneous xenografts. (A and B) HCT116 and SW620 cells, respectively, incubated without primary legumain antibody and stained with Alexa488 labeled rabbit-anti-goat antibody and nuclei stained with DRAQ5™. Scale bars represent 10 µm. (C and D) Hematoxilin/Eosin staining of subcutaneus xenografts from HCT116 and SW620 cells, respectively. Scale bars represent 50 µm. (E and F) Goat-IgG isotype control staining of subcutaneus xenografts from HCT116 and SW620 cells, respectively. A very faint, diffuse background staining was observed in tumor cells, while this was more pronounced in certain areas, possibly necrotic tissue. Scale bar represents 50 µm. (G) Goat-IgG isotype control staining of human colorectal tumor tissue. Scale bar represents 50 µm. (H and I) HCT116 cells incubated with buffers for in situ legumain activity without presence of the cleavable substrate (H) and with substrate and 100 nM recombinant cystatin E/M (I). Scale bars represent 10 µm. (J) Subcutaneous xenografts from SW620 cells incubated with and without substrate, and with substrate and E64 or cystatin E/M. Scale bar represents 200 µm. (TIF) [file pone.0052980.s003.tif]

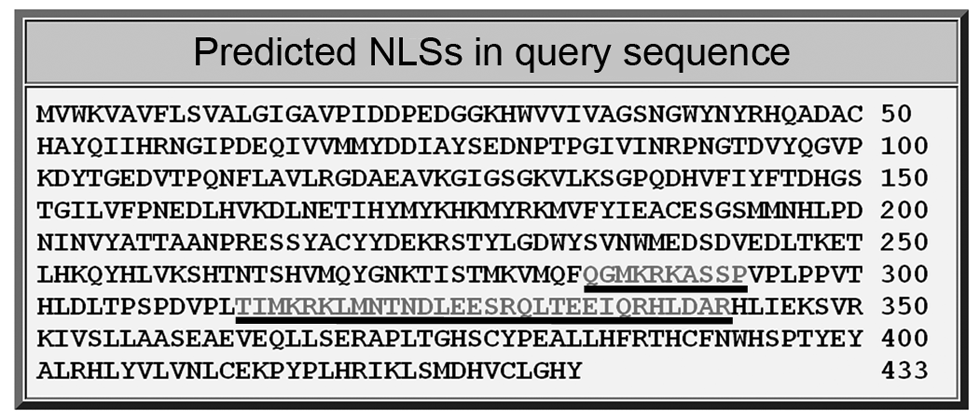

Supplement: Figure S4 — Predicted NLS in legumain. The FASTA sequence of legumain (Q99538) was analyzed for potential NLS signals using NLS-mapper. The return score for monopartite (aa284–293) and bipartite signals (aa313–342) of 6 and 5.1, respectively, indicated a moderately strong, but not exclusive, nuclear localization signal in the protein. (TIF) [file pone.0052980.s004.tif]
